# Supplementary material for: Extraperitoneal Robotic Laparo-Endoscopic Single-Site Plus1-Port Radical Prostatectomy Using the da Vinci Single-Site Platform
Source: J Clin Med. 2021 Apr 8;10(8):1563. doi: 10.3390/jcm10081563 (PMC8068145; doi:10.3390/jcm10081563)
Supplement: Supplementary file 1 [file jcm-10-01563-s001.zip › Supplementary Table 1.docx]

**Supplementary Table 1.** Preoperative, intraoperative and postoperative data on patients who underwent surgery.

| **Parameter** | **Single-site**  **(N = 120)** | | **Multiport**  **(N = 54)** | **p value** |
| --- | --- | --- | --- | --- |
| Age at RP, Mean(range) | 67.1(49.0-78.0) | 64.8(53.0-79.0) | | 0.326 |
| PSA level before RP(ng/mL)  Robotic console time(mins), Mean(range)  Operation time(mins), Mean(range)  pT stage before RP, n(%) | 24.9(18.8-33.8)  140.1(54.0-270.0)  243.1(115.0-440.0) | 20.8(2.3-208.0)  71.2(32.0-135.0)  198.2(105.0-540.0) | | 0.280  ***<0.001***  ***<0.001*** |
| pTx  pT2  pT3  pGleason score at RP, n(%)  6 (grade group 1)  7 (grade group 2-3)  8-10 (grade group 4)  Lymph node dissection  Prostate weight(g) | 3(3%)  68(57%)  49(41%)  46(38%)  45(38%)  29(24%)  61(51%)  41(33-54) | 1(2%)  29(54%)  24(44%)  12(22%)  25(46%)  17(31%)  32(59%)  36 (22-61) | | 0.792  0.716  0.655  ***0.037***  0.274  0.311  0.303  ***0.006*** |
| Positive margin, n (%) | 43(36%) | 23(43%) | | 0.395 |
| Lymph node yield, Mean(range) | 4.8(2-8) | 6.2(3-10)) | | 0.211 |
| Length of stay (days), Mean (range) | 9.1 (5.0-17.0) | 10.2(3.0-16.0) | | 0.882 |
| Detectable PSA after RP, n (%) | 17(14%) | 6(11%) | | 0.582 |
| Incontinence rate, n (%) | 2(1%) | 1(2%) | | 0.931 |
